# Glucose- and glutamine-fueled stabilization of C-Myc is required for T-cell proliferation and malignant transformation

**DOI:** 10.1038/cddiscovery.2016.47

**Published:** 2016-06-27

**Authors:** SS Gabriel, A Kallies

**Affiliations:** 1 The Walter and Eliza Hall Institute of Medical Research, 1G Royal Parade, Parkville, Victoria 3052, Australia; 2 The Department of Medical Biology, University of Melbourne, Parkville, Victoria 3010, Australia

Dynamic regulation of cellular metabolism in response to stimulation or changes in the environment has a crucial role in many biological processes. In particular, T cells undergo dramatic changes in their metabolism when progressing from quiescence to proliferation and acquisition of effector function. This is associated with augmented flux of glucose and glutamine into cells, and is of fundamental importance to meet the increased energy demands associated with growth, proliferation, migration and effector molecule production. Importantly, a similar metabolic switch occurs in cancer cells during malignant transformation. In a study published recently in *Nature Immunology*, Cantrell *et al.* uncover another pathway that is tightly dependent on nutrient availability and critical for T cells.^1^ The authors show that increased and sustained import of glucose and glutamine into activated T cells is indispensable for O-GlcNAcylation, the enzymatically-mediated transfer of N-acetylglucosamine (GlcNAc) to proteins. Multiple intracellular proteins were found to be O-GlcNAcylated in response to T-cell receptor (TCR) signaling, including c-Myc, an important positive regulator of cellular metabolism and proliferation.^1^ O-GlcNAcylation resulted in stabilization of c-Myc, and thus revealed an unappreciated positive-feedback loop between maintenance of cellular metabolism and proliferation that is required for T-cell development, clonal expansion and malignant transformation (Figure 1).

Naive T cells utilize fatty acid oxidation and low levels of glycolysis to sustain survival and homeostatic turnover. In a highly efficient process, pyruvate, the end product of glycolysis, is oxidized in the tricarboxylic acid cycle (TCA) and ATP is produced via oxidative phosphorylation. Triggering of the TCR results in a rapid switch from catabolic to anabolic metabolism, which is characterized by vastly increased rates of aerobic glycolysis and the conversion of glucose-derived pyruvate to lactate. This process is known as the Warburg effect and is a common trait of all rapidly proliferating cells.^2^ A network of transcription factors coordinates this metabolic switch by regulating the expression and activity of enzymes and transporters involved in various metabolic pathways.^3,4^ The transcription factor c-Myc downstream of TCR signaling is a central and non-redundant player in the initial metabolic rewiring to aerobic glycolysis and thereby essential for activation-induced glycolysis and glutaminolysis, as well as cell growth and proliferation.^5^ In addition to transcriptional changes, post-translational protein modifications control aspects of cellular metabolism. The covalent linkage of the monosaccharide GlcNAc to nuclear and cytosolic proteins, a process called O-GlcNAcylation, represents a form of post-translational modification similar to phosphorylation.^6^ The importance of O-GlcNAcylation as a central process in translating nutrient availability into metabolic activity has become evident in various physiological and pathophysiological settings in the past years. For example, diabetic hyperglycemia aggravates insulin-resistance via O-GlcNAcylation of proteins involved in insulin signaling.^7^

O-GlcNAcylation of proteins is controlled by the hexamine biosynthetic pathway (HBP). Although most glucose is transformed to pyruvate in the glycolytic pathway and either excreted as lactate or further metabolized in the TCA, a small amount of fructose-6-phosphate (an intermediate of glycolysis) is branched off into the HBP. In addition to glucose, the HBP requires glutamine, acetyl-CoA, uridine and ATP to generate its high-energy end product uridine diphosphate (UDP)-GlcNAc.^6^ High availability of nutrients is directly reflected in elevated HBP flux and highly increased levels of UDP-GlcNAc. O-GlcNAc transferase (OGT) uses UDP-GlcNAc as donor substrate to modify a wide array of proteins and thereby influences their activity, stability or phosphorylation status. As such, OGT is regarded as 'control center' or 'nutrient sensor' that links nutrient availability to many cellular processes.^8^

The role of O-GlcNAcylation has been extensively studied in cancer cells. Most cancer cells consume high amounts of glucose and glutamine, which together drive hyper-O-GlcNAcylation, a characteristic feature of many transformed cells. O-GlcNAcylation targets glycolytic enzymes and transcription factors that sustain aerobic glycolysis and rapid proliferation, and c-Myc is a well-studied target in this context.^9^ Although phosphorylation of c-Myc at threonine (Thr)-58 causes its rapid degradation, O-GlcNAcylation at the same residue results in stabilization of the protein (Figure 1). Thus, in nutrient-rich environments, O-GlcNAcylation outcompetes phosphorylation at Thr-58 and thereby prevents c-Myc degradation.^10^

Despite its central role in cancer cell metabolism, little was known about the role of O-GlcNAcylation in T cells. Cantrell *et al.* have filled this gap by systematically investigating the dynamics and functional implications of protein O-GlcNAcylation in T cells during development, activation and transformation.^1^ The authors show that activated T cells, including cytotoxic T cells and Th1 cells, consumed massive amounts of glucose and glutamine, which resulted in the generation of high intracellular concentrations of UDP-GlcNAc and O-GlcNAcylation of multiple proteins. In accordance with the notion that high-affinity TCR stimulation directly translates into increased glucose uptake and aerobic glycolysis rate,^11^ this process was dependent upon TCR stimulation time, peptide concentration and the affinity of receptor-ligand interactions. Overall, these data demonstrated that O-GlcNAcylation was a dynamically regulated process in T cells. In the next series of experiments, the authors examined the consequences of genetic loss of OGT during different stages of thymic T-cell development. Strikingly, thymocyte development was almost completely blocked regardless whether OGT was deleted before *β*-selection in double-negative thymocytes or before positive selection in double-positive thymocytes. Importantly, GlcNAcylation was not required for survival or differentiation, but for proliferation during development. In line with this finding, OGT-deficient thymocytes did not undergo malignant transformation in a mouse model of T-cell acute lymphoblastic leukemia. Similar to thymocytes, loss of OGT expression in mature T cells prevented population expansion after TCR stimulation. Remarkably, although many signaling pathways remained intact upon OGT deletion in T cells, including cytokine-mediated STAT phosphorylation and mTOR activity, c-Myc protein expression was prominently reduced. This was in line with the observation that lack of O-GlcNAcylation of c-Myc results in rapid degradation of this transcription factor^10^ and underlines the importance of c-Myc in the early activation phase of T cells. However, this and other studies have shown that O-GlcNAcylation occurs in a plethora of proteins. Therefore, it would be important to examine whether O-GlcNAcylation also affects T-cell function, such as the production of cytotoxic molecules or cytokines. This would be particularly important when considering OGT as a potential therapeutic target. Furthermore, it would be interesting to investigate whether OGT in its role as 'nutrient sensor' is also involved in post-translational modifications of other transcription factors that may directly regulate effector and memory T-cell differentiation.

In summary, the study by Cantrell *et al.* sheds light on a previously unappreciated role of glycolytic metabolism in T cells. Besides being critical for the anabolic metabolism of proliferating T cells and their effector functions, glycolysis is also required for stabilizing c-Myc, which itself is a critical regulator of aerobic glycolysis. Thus, O-GlcNAcylation generates a positive-feedback loop that efficiently sustains cellular metabolism and proliferation (Figure 1). These findings add an additional layer of complexity to the signaling network that controls metabolic adaptation upon immune activation and guides the differentiation of effector T cells.

## Figures and Tables

**Figure 1:**
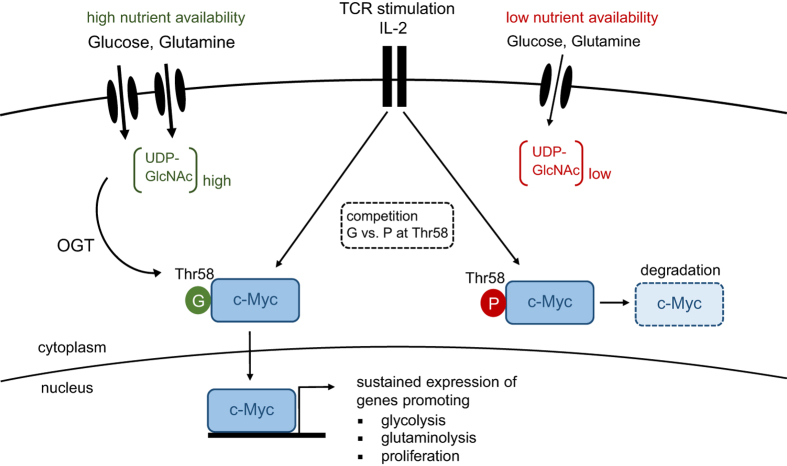
TCR activation and IL-2 signaling results in c-Myc expression, which coordinates the metabolic switch from oxidative phosphorylation to aerobic glycolysis. This process is accompanied by an increased flux of glucose and glutamine into the cell, resulting in elevated levels of UDP-GlcNAc, the end product of the hexamine biosynthetic pathway (HBP). O-GlcNAc transferase (OGT) covalently links GlcNAc sugars to proteins. Under nutrient-rich conditions, O-GlcNAcylation of Thr-58 in c-Myc outcompetes phosphorylation at the same residue, thereby preventing its degradation. Stable c-Myc expression is required for maintenance of aerobic glycolysis and proliferation upon T-cell activation. G, O-GlcNAcylation; P, phosphorylation.

## Notes

SSG and AK are employed at The Walter and Eliza Hall Institute of Medical Research, University of Melbourne, Parkville, Australia.
